# Association between blood urea nitrogen-to-creatinine ratio and mortality risk in critically ill patients with ventilator-associated pneumonia: a retrospective study

**DOI:** 10.3389/fmed.2026.1887689

**Published:** 2026-08-28

**Authors:** Lei Liu, Qiuli He, Muhuo Ji, Jun Liu

**Affiliations:** 1 Department of Emergency and Critical Care Medicine, The Affiliated Suzhou Hospital of Nanjing Medical University, Suzhou Municipal Hospital, Suzhou Clinical Medical Center of Critical Care Medicine, Gusu School of Nanjing Medical University, Suzhou, China; 2 Department of Anesthesiology, The Second Affiliated Hospital of Nanjing Medical University, Nanjing, China

**Keywords:** blood urea nitrogen-to-creatinine ratio, critically ill patients, mortality, RCS, ventilator-associated pneumonia

## Abstract

**Background:**

Ventilator-associated pneumonia (VAP) is a frequent complication of mechanical ventilation that increases mortality and strains healthcare resources. The blood urea nitrogen-to-creatinine ratio (UCR) is a readily available marker of renal function and hydration status. We investigated the association between UCR and short-term mortality in critically ill patients with VAP.

**Methods:**

This retrospective cohort study used data from the MIMIC-IV database and included 2,037 patients with VAP admitted to the intensive care unit (ICU). Patients were categorized into three groups according to tertiles of the blood urea nitrogen-to-creatinine ratio (UCR; T1–T3). Baseline characteristics, laboratory findings, comorbidities, and clinical outcomes were collected within the first 24 h after ICU admission. The primary outcomes were all-cause mortality at 30 and 60 days post-admission. Kaplan–Meier survival analysis and multivariable Cox regression were used to assess the relationship between UCR and mortality, and a restricted cubic spline analysis was performed to explore non-linear associations.

**Results:**

The median UCR among the 2,037 patients was 22.09. Kaplan–Meier analysis showed significant differences in survival across UCR tertiles (log-rank *p* < 0.05), with the highest mortality in the highest tertile (T3). In multivariable Cox regression, after full adjustment the highest tertile (T3) remained independently associated with increased 30-day mortality (HR 1.63, 95% CI 1.14–2.33) relative to the middle tertile (T2); the risk for the lowest tertile (T1) was significant after adjustment for demographics and comorbidities (HR 1.41, 95% CI 1.03–1.94). Restricted cubic spline analysis revealed a non-linear, U-shaped association between UCR and mortality, suggesting a threshold effect.

**Conclusion:**

In the primary cohort, the highest UCR tertile remained associated with higher 30-day mortality after full adjustment, whereas the lowest tertile did not. In the sensitivity model additionally conditioning on first-24-h KDIGO stage and non-renal SOFA, T3 remained associated with mortality (HR 1.68, 95% CI 1.28–2.22), while T1 did not (HR 1.18, 95% CI 0.87–1.60). Adding UCR did not significantly improve cross-validated discrimination beyond SOFA (ΔAUC 0.021, 95% CI −0.004 to 0.045) or SAPS II (ΔAUC −0.001, 95% CI −0.015 to 0.014). UCR should therefore be interpreted as an associative risk marker rather than a causal or treatment target.

## 1 Introduction

Ventilator-associated pneumonia (VAP) is defined as a novel infection of the pulmonary parenchyma that arises in individuals who have undergone endotracheal intubation or tracheostomy. This condition typically manifests within 48 h following the commencement of mechanical ventilation and may persist up to 48 h post-extubation (1). VAP constitutes a significant subtype of hospital-acquired pneumonia (HAP). It is a common and serious complication observed in patients undergoing mechanical ventilation within the intensive care unit (ICU), marked by elevated mortality rates and a considerable strain on healthcare resources (2). VAP demonstrates substantial variability in reported incidence, ranging from 5 to 40%, which is contingent upon the setting and diagnostic criteria employed (3, 4). Similarly, the mortality rate of VAP varies considerably across studies. The mortality rate is approximately 13% in the United States, while a prospective multicenter observational study in Europe has shown that the 30-day mortality rate can reach 30% (5). The occurrence of VAP is closely linked to various risk factors, including steroid therapy, supine positioning, coma, or altered states of consciousness (6, 7). Moreover, the microbial characteristics significantly influence the development of VAP, further intensifying the severity of this condition and the intricacies of treatment (8, 9). VAP considerably affects patient outcomes within the realm of critical care medicine, presenting a significant challenge in clinical management (10–14).

Creatinine, a product of muscle metabolism, is generated at a rate closely linked to an individual's muscle mass. As a result, creatinine concentrations are notably affected by various determinants such as age, gender, and body mass (15). Creatinine is not only a vital biomarker for assessing renal function but also as a sensitive indicator of glomerular filtration rate (GFR). Clinically, therefore, creatinine levels are frequently utilized to assess the renal function status of patients (16). Blood urea nitrogen (BUN), a key byproduct of protein metabolism, is affected by various factors such as protein consumption, hepatic function, and the capacity of the kidneys to excrete waste (17). In clinical settings, BUN is extensively utilized as a marker for evaluating renal function. Research has indicated that BUN has a strong correlation with tubular function, and variations in its levels may indicate alterations in renal filtration and excretion capabilities (18).

The ratio of BUN to creatinine (UCR) is commonly applied in clinical practice to evaluate both renal function and hydration status. The determination of this ratio is quite straightforward, generally achieved by calculating the ratio of BUN concentration (measured in mg/dL) to the concentration of Cr (also expressed in mg/dL) (19). In a study on gastrointestinal bleeding, the UCR was found to be closely associated with the patient's disease status and demonstrated greater value in early assessment compared to traditional renal function indices such as serum creatinine (20). When the UCR increases, it typically indicates a reduction in body fluid volume, potentially caused by dehydration or hypovolemia (21). Conversely, a decrease in the UCR may suggest renal dysfunction or other metabolic abnormalities (22). In individuals suffering from chronic heart failure, variations in the UCR have been shown to be linked to the likelihood of experiencing detrimental clinical outcomes. Notably, a significant relationship has been identified between elevated UCR values and a rise in mortality rates (23, 24). In summary, the UCR functions as an essential indicator for assessing dehydration and volume reduction, a fact supported by a multitude of clinical research findings. Furthermore, it acts as an autonomous prognostic factor for adverse outcomes in critically ill individuals suffering from various conditions including stroke, acute pancreatitis, and acute myocardial infarction (19, 25, 26). Subsequent research could investigate the potential applications of this ratio across various patient demographics, thereby offering more accurate guidance for clinical practice (27).

Beyond its general use as a marker of renal function and volume status, several lines of evidence suggest that UCR may be mechanistically linked to outcomes in VAP specifically. First, UCR is a sensitive index of hydration: dehydration impairs airway mucociliary clearance and alters the rheology of airway secretions, weakening the primary mechanical barrier against pulmonary infection. Second, hydration status modulates innate immunity—serum osmolality has been inversely associated with pro-inflammatory cytokine production, indicating that volume depletion may dysregulate the immune response to pulmonary pathogens (28, 29). Third, an elevated UCR often reflects a hypercatabolic, high-protein-turnover state and prerenal hypoperfusion, both common in severe sepsis and capable of aggravating organ injury in VAP. Conversely, a low UCR may signal intrinsic renal dysfunction, fluid overload, or reduced muscle mass and frailty, each independently associated with poor prognosis in the critically ill. These opposing derangements provide an *a priori* rationale for hypothesizing a non-linear, U-shaped relationship in which both abnormally high and abnormally low UCR values predict increased mortality in VAP.

Considering the identified research gaps and previous discoveries, our investigation was structured to explore the connection between UCR and mortality outcomes in critically ill individuals diagnosed with VAP. Consequently, we aimed to perform a comprehensive retrospective cohort study leveraging the MIMIC-IV database to scrutinize the link between UCR and all-cause mortality at both 30 and 60 days within this critically ill group of VAP patients. The findings from our study could provide valuable insights into potential biomarkers that may enhance early risk stratification in this susceptible population (30).

## 2 Materials and methods

### 2.1 Data sources and ethics declarations

The dataset employed in this study was sourced from the MIMIC-IV (version 3.1) database, which is a publicly accessible and extensive resource created by the Computational Physiology Laboratory at the Massachusetts Institute of Technology. This database contains detailed records for more than 90,000 patients who were admitted to the ICU at Beth Israel Deaconess Medical Center (BIDMC) during the period from 2008 to 2022. It includes a diverse array of data types, encompassing laboratory results, medication administration, and other relevant clinical information (31). The MIMIC-IV (v 3.1) significantly improves the accuracy and completeness of data by addressing existing data challenges, thus providing a more dependable large-scale public database for clinical research. This enhancement plays a crucial role in facilitating disease prediction, evaluating treatment efficacy, and conducting various analyses, ultimately propelling advancements within the medical field. All personal identifiers have been anonymized, with actual patient data substituted with randomly generated numbers. As a result, the necessity for obtaining ethical approval and informed consent was eliminated. One of the authors met the criteria for database access and performed the data extraction, guaranteeing adherence to the ethical standards that regulate the utilization of research data.

### 2.2 Study population

This investigation centered on individuals identified with Ventilator-Associated Pneumonia (VAP), where the diagnosis was established utilizing ICD-9 codes (4957 and 99731) and the ICD-10 code (J95851)3. The criteria for exclusion included: (1) individuals who had previous admissions to the Intensive Care Unit (ICU); (2) a hospital duration of less than 24 h; (3) failure to satisfy the diagnostic criteria for VAP; and (4) incomplete data regarding Blood Urea Nitrogen (BUN) and Creatinine (Cr) levels. In total, 2037 patients were included in this analysis. The participants were classified into three distinct groups based on the tertiles of blood urea nitrogen-to-creatinine ratio (UCR) levels: T1: 11.32 ± 2.72; T2: 19.05 ± 2.34; T3: 35.87 ± 16.28.

### 2.3 Data collection

Within the first 24 h after ICU admission, we collected demographic variables, vital signs, BUN, serum creatinine, UCR, hematologic and biochemical measurements, comorbidities, medication exposures, organ-support interventions, and severity scores. For all 2,037 patients in the original analytic cohort, we derived maximum first-24-h KDIGO AKI stage from serum creatinine change relative to the lowest prior 48-hour and 7-day values, weight-normalized urine-output rates over 6, 12, and 24 hours, and CRRT events, following the KDIGO 2012 criteria and the current MIT-LCP MIMIC-IV implementation (32). Complete SOFA scores and their six organ components were linked by stay_id and checked against the original analytic file. First-day minimum albumin (MIMIC item 50862, g/dL) was available for 513 patients and was used only in a complete-case sensitivity analysis.

### 2.4 Expose variable and clinical outcome

In this study, the exposure variable was the UCR. To reduce treatment-related interference, BUN and Cr values were taken from the first laboratory tests after admission. The primary outcomes were all-cause mortality at 30 and 60 days following ICU admission. These two time points were chosen to capture both the acute phase of VAP-related mortality (30-day), during which most ventilator-associated deaths occur, and the broader short-term window (60-day) that reflects the protracted clinical course of mechanically ventilated critically ill patients, whose mean hospital stay in this cohort exceeded 4 weeks. A 30-day endpoint alone would prematurely censor a substantial proportion of patients still hospitalized, whereas the 60-day endpoint captures the full short-term mortality burden without extending into the longer-term period dominated by competing chronic comorbidities. The 30-day endpoint simultaneously addresses early, clinically meaningful mortality.

### 2.5 Statistical analysis

The cohort in this investigation was divided into three distinct groups according to the tertiles of the UCR. Descriptive statistics were generated for the entire population, while inferential statistical methods were implemented to confirm the validity and reliability of the results. Categorical data were presented as counts in conjunction with their corresponding percentages. For continuous variables conforming to a normal distribution, values were reported as means accompanied by standard deviations (SD). In contrast, for those variables demonstrating a non-normal distribution, the median and interquartile range (IQR) were employed. The chi-square test was utilized for categorical variables, the *t*-test was applied for continuous variables with normal distribution, and the Kruskal-Wallis test was used for continuous variables that did not follow a normal distribution to enable statistical comparisons.

Survival analyses for 30-day and 60-day outcomes were performed using Kaplan-Meier curves. Cox proportional hazards models estimated hazard ratios (HRs) and 95% CIs for 30-day mortality, using T2 as the reference. Model 1 adjusted for age, sex, BMI, height, and weight. Model 2 additionally adjusted for vital signs and major comorbidities including renal disease. Model 3 further included laboratory indices, Charlson Comorbidity Index, SAPS II, SOFA, GCS, vasopressin, norepinephrine, ACEI/ARB, thiazide diuretics, NSAIDs, hospital stay, and mechanical ventilation. Reviewer-requested renal sensitivity models sequentially adjusted for age and sex, comorbidities, KDIGO stage, and non-renal SOFA. Because KDIGO and UCR share serum creatinine, KDIGO-conditioned models were labeled alternative-estimand analyses. Total SOFA, SAPS II, and the separate CRRT indicator were omitted from the final renal sensitivity model because their renal, urea, or renal-replacement components overlap with UCR or KDIGO; non-renal SOFA was defined as total SOFA minus its renal component. A separate complete-case model additionally included first-day minimum albumin and, because only 77 events were available among 513 patients, used the more parsimonious adjustment set of age, sex, KDIGO, and non-renal SOFA. Incremental discrimination was assessed for SOFA vs. SOFA plus UCR and SAPS II vs. SAPS II plus UCR using log_2_(UCR) and its squared term, 20 repeats of stratified 10-fold cross-validation, and 2,000 paired stratified bootstrap samples for AUC differences. Decision-curve analysis used the cross-validated probabilities. APACHE II was unavailable.

## 3 Results

### 3.1 Baseline characteristics

**Figure 1:**
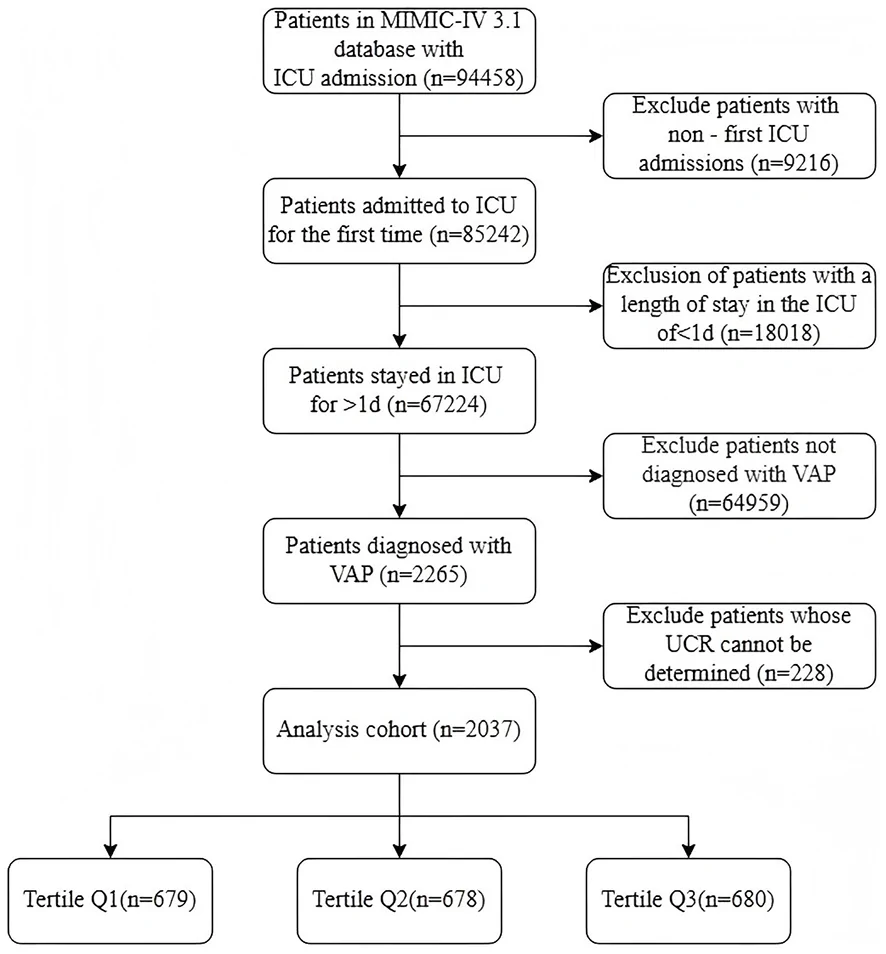
Flow chart for the selection of study participants. ICU, the intensive care unit; MIMIC, the medical information mart for intensive care; VAP, ventilator-associated pneumonia; UCR, the blood urea nitrogen to creatinine ratio.

Initially, a cohort of 2,037 critically ill individuals diagnosed with VAP was identified as eligible for inclusion in this research, adhering to the established inclusion and exclusion criteria (Figure 1). The baseline characteristics of all subjects are categorized based on the tertiles of the admission blood urea nitrogen-to-creatinine ratio (UCR) in Table 1. Within this population, males represented 65.10%, while females accounted for 34.90%. The participants had a mean age of 63.09 years, with the average UCR value being 22.09. Notably, the levels of blood urea nitrogen, creatinine, and UCR exhibited significant variations across the different groups. Furthermore, T1 showed higher serum potassium and lactate levels, the highest creatinine level, the highest proportion of renal disease, and significantly higher usage rates of vasopressors and CRRT. Patients in T3 with higher UCR values had significantly elevated BUN but the lowest creatinine, characterized by advanced age, high comorbidity burden, and the lowest intervention intensity. Although T3 patients had the shortest hospital stay, their 30-day mortality rate was significantly higher than that of T2. Then, compared with T1 and T3, T2 had intermediate creatinine levels, lower age and comorbidity burden, moderate usage rates of vasopressors and CRRT, and intermediate levels of lactate, anion gap, and bicarbonate. Finally, the T2 group (middle UCR tertile) had the lowest 30-day (12.68%) and 60-day (14.31%) mortality rates, representing the population with the best prognosis. Both T1 and T3 groups significantly deviated from T2 in key clinical characteristics (most *p* < 0.05).

**Table 1:** Baseline characteristics of the study population by UCR tertiles.

Variable	Total (*n* = 2,037)	Q1 (*n* = 679)	Q2 (*n* = 678)	Q3 (*n* = 680)	*p*-value
UCR	22.09 ± 14.07	11.32 ± 2.72	19.05 ± 2.34	35.87 ± 16.28	<0.0001
Sex, *n* (%)					0.03
Female	711(34.90)	214(31.52)	237(34.96)	260(38.24)	
Male	1,326(65.10)	465(68.48)	441(65.04)	420(61.76)	
Age, year	63.09 ± 15.94	57.29 ± 16.20	64.77 ± 14.95	67.19 ± 14.97	<0.0001
Height, m	1.70 ± 0.11	1.72 ± 0.10	1.70 ± 0.10	1.69 ± 0.11	<0.0001
Weight, kg	88.08 ± 27.52	91.33 ± 27.64	89.71 ± 28.10	83.22 ± 26.17	<0.0001
BMI, kg/m^2^	30.50 ± 9.13	31.10 ± 9.01	31.08 ± 9.44	29.29 ± 8.84	<0.001
SBP, mmHg	117.40 ± 16.12	117.23 ± 16.74	117.92 ± 16.19	117.06 ± 15.41	0.58
DBP, mmHg	63.09 ± 10.20	64.02 ± 9.96	62.70 ± 10.41	62.57 ± 10.16	0.01
MBP, mmHg	79.04 ± 10.19	79.73 ± 10.31	79.21 ± 10.07	78.19 ± 10.15	0.02
Respiratory rate, beats/min	20.85 ± 4.36	20.74 ± 4.48	20.59 ± 4.30	21.20 ± 4.28	0.03
Temperature, °C	37.01 ± 0.72	37.06 ± 0.74	37.02 ± 0.66	36.96 ± 0.76	0.04
BUN, mg/ml	31.55 ± 24.79	24.87 ± 22.02	29.26 ± 21.77	40.50 ± 27.49	<0.0001
Creatinine, mg/ml	1.72 ± 1.68	2.42 ± 2.41	1.54 ± 1.14	1.21 ± 0.80	<0.0001
Hb, g/L	10.78 ± 2.14	11.02 ± 2.13	10.91 ± 2.07	10.40 ± 2.18	<0.0001
WBC, 10^9^/L	14.09 ± 10.58	13.81 ± 7.45	13.92 ± 11.28	14.57 ± 12.44	0.39
Platelet, 10^9^/L	206.33 ± 102.99	194.07 ± 91.63	205.71 ± 98.32	219.69 ± 116.51	<0.0001
RDW, %	15.39 ± 2.37	15.47 ± 2.49	15.06 ± 2.17	15.65 ± 2.42	<0.0001
Glucose, mg/dL	164.75 ± 122.35	163.38 ± 73.97	162.00 ± 66.27	168.83 ± 186.81	0.55
Sodium, mmol/L	140.05 ± 5.75	139.78 ± 5.71	139.65 ± 5.08	140.71 ± 6.34	<0.01
Potassium, mmol/L	4.40 ± 0.75	4.45 ± 0.82	4.41 ± 0.71	4.32 ± 0.71	<0.01
PT, s	17.46 ± 10.70	17.44 ± 10.68	17.55 ± 11.77	17.39 ± 9.55	0.97
APTT, s	48.34 ± 34.25	49.42 ± 35.07	47.61 ± 33.53	47.99 ± 34.17	0.61
Lactate (max), mg/dl	3.40 ± 3.10	4.12 ± 3.70	3.38 ± 2.91	2.61 ± 2.24	<0.0001
Bicarbonate, mmol/L	23.59 ± 4.82	22.45 ± 4.00	23.22 ± 4.33	25.10 ± 5.60	<0.0001
Anion gap	15.14 ± 4.73	16.49 ± 5.50	14.97 ± 4.06	13.95 ± 4.16	<0.0001
Renal disease, *n* (%)					< 0.001
No	1,554(76.29)	493(72.61)	510(75.22)	551(81.03)	
Yes	483(23.71)	186(27.39)	168(24.78)	129(18.97)	
Myocardial infarction, *n* (%)					0.05
No	1,643(80.66)	568(83.65)	536(79.06)	539(79.26)	
Yes	394(19.34)	111(16.35)	142(20.94)	141(20.74)	
Congestive heart failure, n(%)					< 0.001
No	1,354(66.47)	486(71.58)	450(66.37)	418(61.47)	
Yes	683(33.53)	193(28.42)	228(33.63)	262(38.53)	
Chronic pulmonary disease, *n* (%)					< 0.01
No	1,469(72.12)	514(75.70)	496(73.16)	459(67.50)	
Yes	568(27.88)	165(24.30)	182(26.84)	221(32.50)	
Diabetes, *n* (%)					0.10
No	1,352(66.37)	472(69.51)	443(65.34)	437(64.26)	
Yes	685(33.63)	207(30.49)	235(34.66)	243(35.74)	
Malignant cancer, *n* (%)					0.03
No	1,841(90.38)	630(92.78)	607(89.53)	604(88.82)	
Yes	196(9.62)	49(7.22)	71(10.47)	76(11.18)	
Vasopressin, *n* (%)					< 0.0001
No	1,572(77.17)	486(71.58)	531(78.32)	555(81.62)	
Yes	465(22.83)	193(28.42)	147(21.68)	125(18.38)	
Norepinephrine, *n* (%)					0.01
No	997(48.94)	302(44.48)	354(52.21)	341(50.15)	
Yes	1,040(51.06)	377(55.52)	324(47.79)	339(49.85)	
ACEI.ARB, *n* (%)					0.35
No	1,511(74.18)	515(75.85)	491(72.42)	505(74.26)	
Yes	526(25.82)	164(24.15)	187(27.58)	175(25.74)	
Thiazide.diuretic, *n* (%)					< 0.0001
No	329(16.15)	162(23.86)	82(12.09)	85(12.50)	
Yes	1,708(83.85)	517(76.14)	596(87.91)	595(87.50)	
NSAID, *n* (%)					0.16
No	977(47.96)	334(49.19)	305(44.99)	338(49.71)	
Yes	1,060(52.04)	345(50.81)	373(55.01)	342(50.29)	
CRRT, *n* (%)					< 0.0001
No	1,876(92.19)	600(88.37)	627(92.75)	649(95.44)	
Yes	159(7.81)	79(11.63)	49(7.25)	31(4.56)	
Mechanical ventilation, *n* (%)					0.16
No	80(3.93)	19(2.80)	32(4.72)	29(4.26)	
Yes	1,957(96.07)	660(97.20)	646(95.28)	651(95.74)	
Charlson comorbidity index, *n* (%)	5.06 ± 2.97	4.48 ± 3.08	5.27 ± 2.91	5.44 ± 2.84	<0.0001
SAPS-II	42.92 ± 14.91	42.45 ± 15.71	42.33 ± 14.93	43.98 ± 13.99	0.07
SOFA	6.89 ± 3.96	7.69 ± 4.35	6.56 ± 3.79	6.42 ± 3.57	<0.0001
GCS	13.05 ± 3.67	12.83 ± 3.91	13.21 ± 3.49	13.10 ± 3.59	0.14
los_hospital_day	27.67 ± 21.63	29.67 ± 23.95	28.32 ± 20.94	25.04 ± 19.53	<0.001
los_icu_day	16.98 ± 13.51	18.33 ± 15.03	16.97 ± 12.08	15.63 ± 13.12	<0.01
survival_days_30	15.22 ± 8.95	16.05 ± 8.74	15.55 ± 9.17	14.08 ± 8.82	<0.001
status_yn_30, *n* (%)					< 0.01
Alive	1,728(84.83)	585(86.16)	592(87.32)	551(81.03)	
Death	309(15.17)	94(13.84)	86(12.68)	129(18.97)	
survival_days_60	16.74 ± 12.32	17.87 ± 12.74	16.92 ± 12.00	15.43 ± 12.10	<0.01
status_yn_60, *n* (%)					< 0.01
Alive	1,695(83.21)	573(84.39)	581(85.69)	541(79.56)	
Death	342(16.79)	106(15.61)	97(14.31)	139(20.44)	

Survival analysis of the 2037 included patients demonstrated that the non-survivor group (*n* = 309) exhibited significantly worse clinical baseline status than the survivor group (*n* = 1,728; *p* < 0.01; Table 2). Combined with Table 1, patients in the T2 group not only had the highest 30-day survival rate but also the highest proportion in the total survivor population. The non-survivor group showed significantly higher age, Charlson Index, and SOFA score than the survivor group. Meanwhile, the non-survivors had higher usage rates of vasopressors and mechanical ventilation, but significantly lower ACEI/ARB usage. The average UCR in the non-survivor cohort was significantly elevated compared to that of the survivor cohort. Notably, individuals categorized in the T3 group represented over 41.75% of the non-survivor cohort, which is substantially higher than their representation of 33.38% within the overall population. Conversely, the T2 group exhibited the lowest representation at 27.83% in the non-survivor cohort, aligning with its designation as having the most favorable prognosis.

**Table 2:** Comparison of baseline characteristics between three groups segmented by 30-day mortality.

Variable	Total (*n* = 2,037)	Survival (*n* = 1,728)	Non-survival (*n* = 309)	*p*-value
Age, year	63.09 ± 15.94	62.00 ± 16.19	69.15 ± 12.96	<0.0001
Sex, *n* (%)				0.76
Female	711(34.90)	606(35.07)	105(33.98)	
Male	1,326(65.10)	1,122(64.93)	204(66.02)	
Weight, kg	88.08 ± 27.52	88.35 ± 27.84	86.58 ± 25.73	0.27
BMI, kg/m^2^	30.50 ± 9.13	30.60 ± 9.24	29.99 ± 8.53	0.28
SBP, mmHg	117.40 ± 16.12	117.36 ± 16.02	117.67 ± 16.66	0.76
DBP, mmHg	63.09 ± 10.20	63.38 ± 10.09	61.48 ± 10.64	<0.01
MBP, mmHg	79.04 ± 10.19	79.15 ± 10.15	78.42 ± 10.39	0.25
Respiratory rate, beats/min	20.85 ± 4.36	20.70 ± 4.27	21.65 ± 4.73	<0.01
Temperature, °C	37.01 ± 0.72	37.05 ± 0.69	36.79 ± 0.85	<0.0001
BUN, mg/ml	31.55 ± 24.79	30.15 ± 23.92	39.39 ± 27.96	<0.0001
Creatinine, mg/ml	1.72 ± 1.68	1.68 ± 1.70	1.95 ± 1.56	<0.01
Hemoglobin, g/L	10.78 ± 2.14	10.80 ± 2.13	10.65 ± 2.23	0.30
WBC, 10^9^/L	14.09 ± 10.58	13.84 ± 9.41	15.56 ± 15.61	0.07
Platelet, 10^9^/L	206.33 ± 102.99	207.43 ± 102.43	200.10 ± 106.12	0.28
RDW, %	15.39 ± 2.37	15.33 ± 2.37	15.74 ± 2.36	<0.01
Glucose, mg/dL	164.75 ± 122.35	163.41 ± 129.31	172.30 ± 71.38	0.08
Sodium, mmol/L	140.05 ± 5.75	140.14 ± 5.73	139.50 ± 5.84	0.07
Potassium, mmol/L	4.40 ± 0.75	4.38 ± 0.76	4.51 ± 0.68	<0.01
PT, s	17.46 ± 10.70	17.29 ± 10.80	18.40 ± 10.13	0.09
APTT, s	48.34 ± 34.25	47.32 ± 33.13	53.90 ± 39.46	<0.01
Lactate (max), mg/dl	3.40 ± 3.10	3.33 ± 2.99	3.75 ± 3.60	0.08
Bicarbonate, mmol/L	23.59 ± 4.82	23.63 ± 4.79	23.39 ± 5.02	0.45
Anion gap	15.14 ± 4.73	14.98 ± 4.64	16.02 ± 5.16	<0.01
Renal disease, *n* (%)				< 0.0001
No	1,554(76.29)	1,347(77.95)	207(66.99)	
Yes	483(23.71)	381(22.05)	102(33.01)	
Myocardial infarction, *n* (%)				< 0.001
No	1,643(80.66)	1,417(82.00)	226(73.14)	
Yes	394(19.34)	311(18.00)	83(26.86)	
Congestive heart failure, *n* (%)				< 0.0001
No	1,354(66.47)	1,188(68.75)	166(53.72)	
Yes	683(33.53)	540(31.25)	143(46.28)	
Chronic pulmonary disease, *n* (%)				0.05
No	1,469(72.12)	1,261(72.97)	208(67.31)	
Yes	568(27.88)	467(27.03)	101(32.69)	
Diabetes, *n* (%)				0.03
No	1,352(66.37)	1,164(67.36)	188(60.84)	
Yes	685(33.63)	564(32.64)	121(39.16)	
Malignant cancer, n (%)				0.04
No	1,841(90.38)	1,572(90.97)	269(87.06)	
Yes	196(9.62)	156(9.03)	40(12.94)	
Vasopressin, n (%)				< 0.0001
No	1,572(77.17)	1,387(80.27)	185(59.87)	
Yes	465(22.83)	341(19.73)	124(40.13)	
Norepinephrine, n (%)				< 0.0001
No	997(48.94)	886(51.27)	111(35.92)	
Yes	1,040(51.06)	842(48.73)	198(64.08)	
ACEI.ARB, n (%)				< 0.0001
No	1,511(74.18)	1,238(71.64)	273(88.35)	
Yes	526(25.82)	490(28.36)	36(11.65)	
Thiazide.diuretic, n (%)				0.35
No	329(16.15)	273(15.80)	56(18.12)	
Yes	1,708(83.85)	1,455(84.20)	253(81.88)	
NSAID, n (%)				0.25
No	977(47.96)	819(47.40)	158(51.13)	
Yes	1,060(52.04)	909(52.60)	151(48.87)	
CRRT, n (%)				0.22
No	1,876(92.19)	1,597(92.53)	279(90.29)	
Yes	159(7.81)	129(7.47)	30(9.71)	
Mechanical_Ventilation, n (%)				< 0.01
No	80(3.93)	78(4.51)	2(0.65)	
Yes	1,957(96.07)	1,650(95.49)	307(99.35)	
Charlson comorbidity index	5.06 ± 2.97	4.85 ± 2.95	6.28 ± 2.80	<0.0001
SAPS-II	42.92 ± 14.91	41.86 ± 14.73	48.86 ± 14.52	<0.0001
SOFA	6.89 ± 3.96	6.70 ± 3.93	7.94 ± 3.95	<0.0001
GCS	13.05 ± 3.67	13.11 ± 3.58	12.68 ± 4.12	0.09
Hospital mortality	27.67 ± 21.63	29.99 ± 22.37	14.70 ± 9.36	<0.0001
ICU mortality	16.98 ± 13.51	17.59 ± 14.29	13.56 ± 6.83	<0.0001
survival_days_30	15.22 ± 8.95	15.55 ± 9.24	13.42 ± 6.84	<0.0001
survival_days_60	16.74 ± 12.32	17.33 ± 12.97	13.42 ± 6.84	<0.0001
status_yn_60, n (%)				< 0.0001
Alive	1,695(83.21)	1,695(98.09)		
Death	342(16.79)	33(1.91)	309(100.00)	
UCR	22.09 ± 14.07	21.69 ± 13.96	24.32 ± 14.49	<0.01
UCRQ				< 0.01
Q1	679(33.33)	585(33.85)	94(30.42)	
Q2	678(33.28)	592(34.26)	86(27.83)	
Q3	680(33.38)	551(31.89)	129(41.75)	

### 3.2 Kaplan–Meier survival analysis

**Figure 2:**
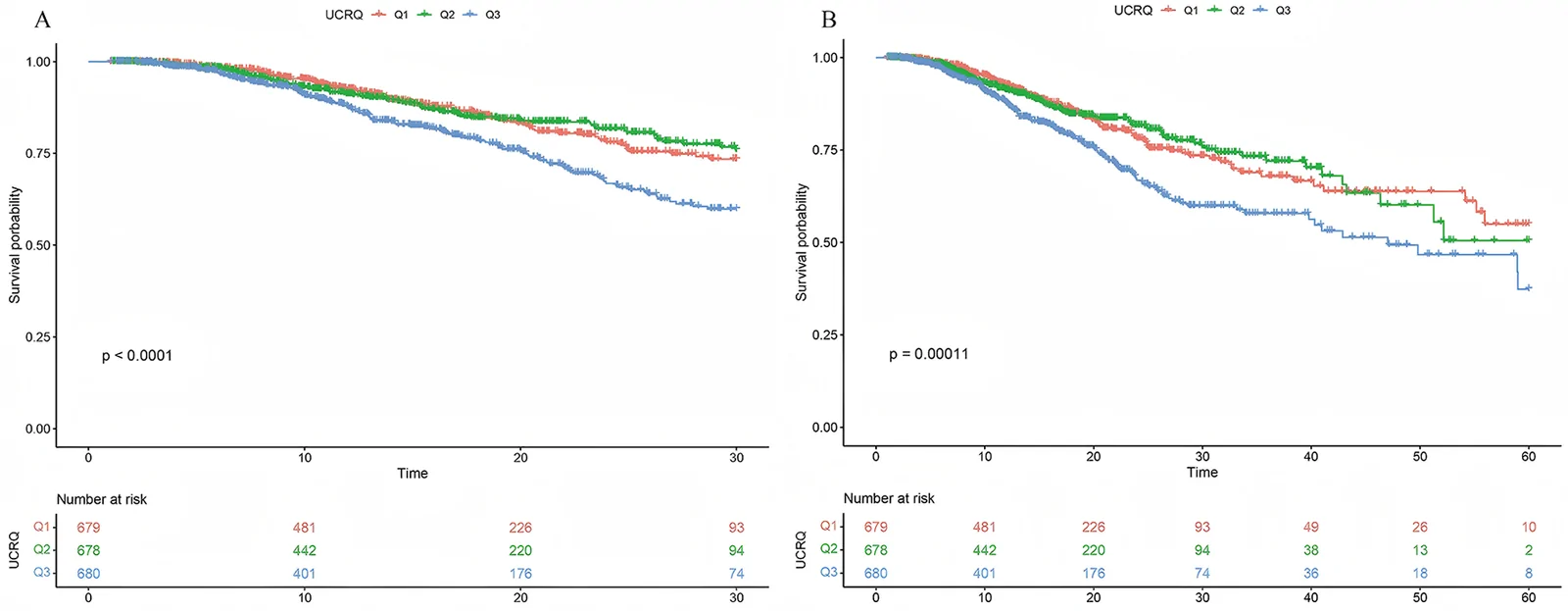
Kaplan–Meier survival analysis curves for all-cause mortality in patients with VAP. **(A)** 30-day mortality; **(B)** 60-day mortality.

Patients in the UCR tertile groups (T1–T3) showed significant differences in 30-day and 60-day survival (log-rank *P* < 0.05), supporting the influence of UCR on prognosis (Figure 2). Survival in the highest-UCR group (T3) declined relatively rapidly, and the separation in survival probability among groups widened over time, with the excess mortality risk of T3 becoming more pronounced during later follow-up.

### 3.3 Cox regression analysis

Multivariable Cox regression was used to assess the association between UCR tertiles and 30-day mortality (Table 3). Relative to T2, T3 was associated with higher mortality in the crude model and remained associated after full adjustment (Model 3: HR 1.63, 95% CI 1.14–2.33; *P* = 0.01). T1 was associated with higher mortality in Model 2 (HR 1.41, 95% CI 1.03–1.94; *P* = 0.03), but this association was attenuated in Model 3 (HR 1.14, 95% CI 0.79–1.65; *P* = 0.49). Because the prespecified interpretation was non-linear and T2 was the reference, the linear trend statistic was not used to support a monotonic dose-response claim.

**Table 3:** Multivariable Cox regression to assess the association between UCR and 30-day mortality.

Character	Crude model		Model 1		Model 2		Model 3	
	95% CI	*P*	95% CI	*P*	95% CI	*P*	95% CI	*P*
survival_days_30, status_01_30 ~UCRQ								
Q2	Ref		Ref		Ref		Ref	
Q1	1.05(0.79,1.41)	0.73	1.32(0.97, 1.79)	0.08	1.41(1.03, 1.94)	0.03	1.14(0.79, 1.65)	0.49
Q3	1.72(1.31,2.26)	<0.001	1.53(1.15, 2.04)	0.004	1.5(1.11, 2.03)	0.01	1.63(1.14, 2.33)	0.01
*p* for trend (character2integer)		<0.0001		0.004		0.01		0.01

HR, hazard ratio; 95 % CI, 95 % confidence interval; Ref, reference. Model 1: UCRQ, age, sex, BMI, height, weight; model 2: UCRQ, age, sex, BMI, height, weight, SBP, DBP, MBP, respiratory rate, temperature, renal disease, myocardial infarction, congestive heart failure, chronic pulmonary disease, diabetes, malignant cancer; model 3: UCRQ, age, sex, BMI, height, weight, SBP, DBP, MBP, respiratory rate, temperature, renal disease, myocardial infarction, congestive heart failure, chronic pulmonary disease, diabetes, malignant cancer, lactate max, PT, APTT, anion gap, bicarbonate, glucose, sodium, potassium, Charlson comorbidity index, sapsii, sofa, GCS, vasopressin, norepinephrine, ACEI.ARB, Thiazide.diuretic, NSAID, long title, mechanical ventilation.

### 3.4 Restricted cubic spline analysis

**Figure 3:**
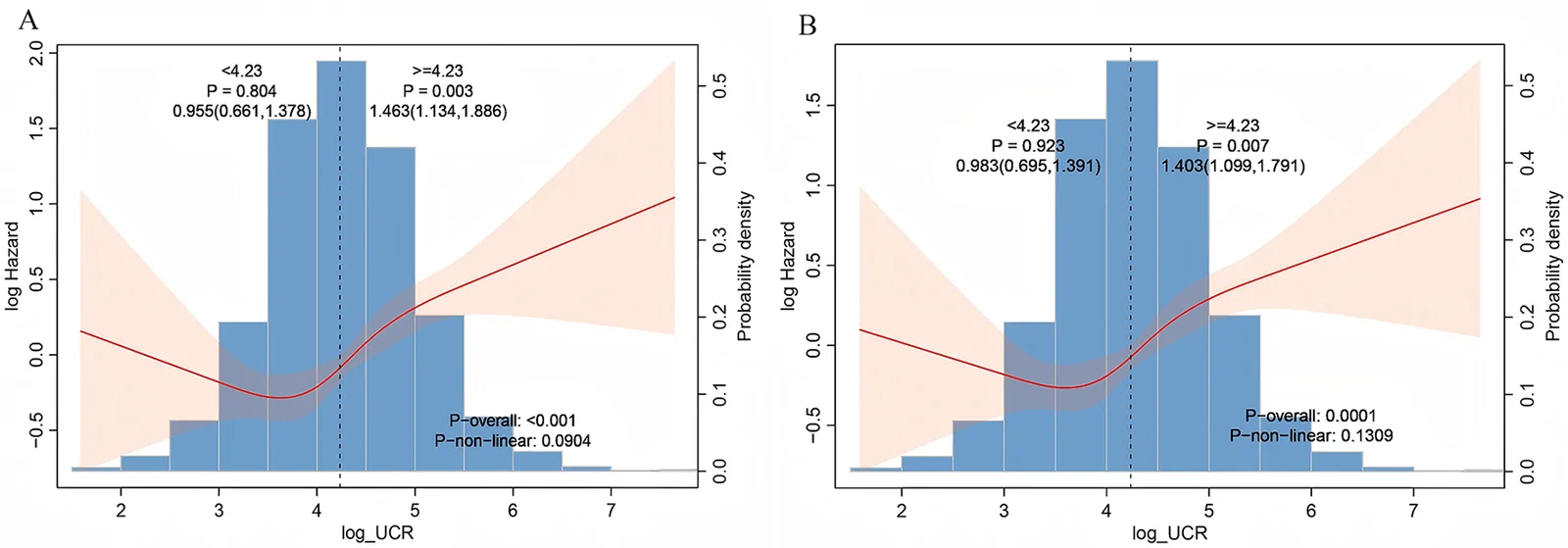
Restricted cubic spline plot for all-cause mortality. Heavy central lines represent the estimated adjusted hazard ratios, with shaded ribbons denoting 95% confidence intervals. **(A)** 30-day mortality; **(B)** 60-day mortality.

Using a RCS model, we investigated the nonlinear relationship between the logarithm of the UCR and the outcomes at 30 and 60 days in patients (Figure 3). The impact of UCR levels on patient prognosis was not linearly cumulative, but rather suggested a critical threshold: the effect on prognosis remained relatively minor within a specific threshold range, whereas the risk increased significantly beyond this threshold.

### 3.5 Subgroup analyses of UCR in VAP patients

**Figure 4:**
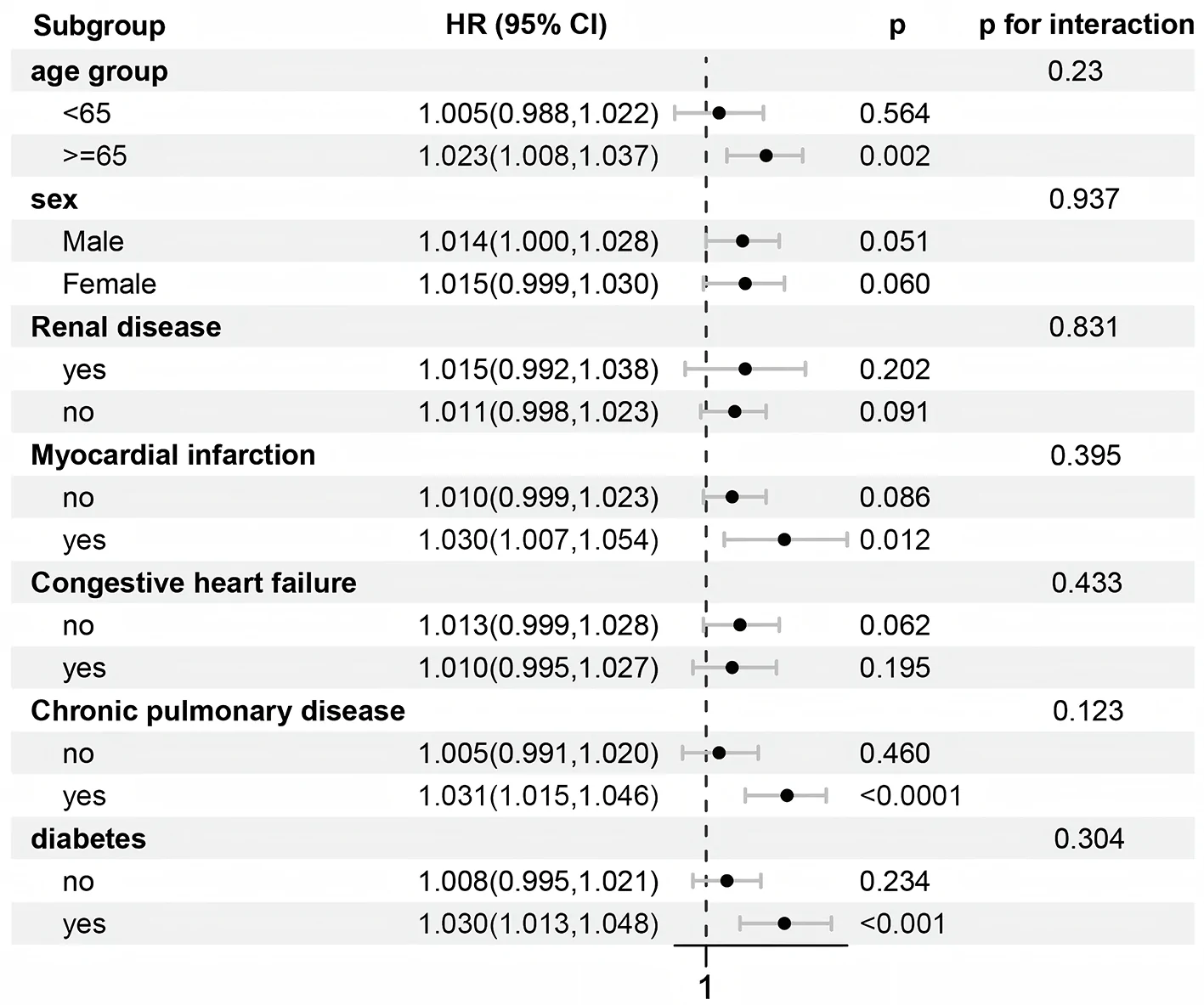
Subgroup analysis for the association between UCR and mortality in critically ill patients with VAP.

To investigate the correlation between the UCR and the mortality risk among patients diagnosed with VAP, subgroup analyses were performed to evaluate the differential impact of UCR on mortality across various clinical characteristic cohorts (Figure 4). No statistically significant interactions were observed in subgroups classified by age, gender, renal impairment, myocardial infarction, congestive heart failure, chronic pulmonary disease, or diabetes (*P* for interaction >0.05).

### 3.6 Sensitivity analyses

All 2,037 original cohort stays matched the local MIMIC-IV 3.0 source by subject_id, hadm_id, stay_id, and ICU admission time. Maximum first-24-h KDIGO stage was 0 in 706 patients, 1 in 390, 2 in 714, and 3 in 227; corresponding 30-day mortality was 11.3%, 14.1%, 16.9%, and 23.3%. Creatinine criteria were evaluable in 2,030 patients, at least 6 h of urine-output criteria in 1,904, and first-day CRRT was identified in 73. In the model adjusting for age, sex, comorbidities, KDIGO stage, and non-renal SOFA, T3 remained associated with higher mortality relative to T2 (HR 1.68, 95% CI 1.28–2.22; *P* < 0.001), whereas T1 did not (HR 1.18, 95% CI 0.87–1.60; *P* = 0.290). In the albumin complete-case model (*n* = 513; 77 events), T3 remained associated with mortality (HR 2.52, 95% CI 1.39–4.54; *P* = 0.002), whereas T1 did not (HR 1.53, 95% CI 0.80–2.93; *P* = 0.202). These KDIGO-conditioned estimates are alternative estimands because UCR and KDIGO share serum creatinine; the albumin result is additionally subject to selection from incomplete testing.

For 30-day mortality, the repeated cross-validated AUC was 0.580 (95% CI 0.544–0.614) for SOFA and 0.601 (95% CI 0.568–0.634) for SOFA plus UCR. The paired ΔAUC was 0.021 (95% CI −0.004 to 0.045; *P* = 0.087). The corresponding AUC was 0.640 (95% CI 0.608–0.672) for SAPS II and 0.640 (95% CI 0.607–0.671) after adding UCR (ΔAUC −0.001, 95% CI −0.015 to 0.014; *P* = 0.924). Decision curves showed no consistent separation between either score and its UCR-extended model. Thus, these analyses do not establish incremental clinical utility. Supplementary Tables S1–S4 and Supplementary Figures S1, S2 provide the verification, renal sensitivity, and performance results.

## 4 Discussion

In this retrospective cohort of 2,037 critically ill patients with VAP from the MIMIC-IV database, UCR was associated with short-term mortality and the pattern was non-linear. T2 had the lowest observed 30-day (12.68%) and 60-day (14.31%) mortality. After full adjustment, T3 remained associated with higher 30-day mortality (HR 1.63, 95% CI 1.14–2.33), whereas the estimate for T1 was attenuated and no longer statistically significant (HR 1.14, 95% CI 0.79–1.65). The spline analysis was consistent with non-linearity, but neither the tertile analysis nor the spline establishes a universal optimal UCR interval or a causal treatment target.

Several mechanisms may explain these findings. A high UCR can reflect dehydration or prerenal hypoperfusion, but it may also arise from gastrointestinal bleeding, increased protein intake, or severe catabolism. Dehydration may impair airway mucociliary clearance and alter immune responses, whereas a hypercatabolic state may increase urea generation independently of hydration. Conversely, a low UCR can reflect intrinsic renal dysfunction, fluid overload, reduced muscle mass, or frailty. In our cohort, T1 had the highest creatinine and greater vasopressor and CRRT use, whereas T3 combined high BUN with the lowest creatinine, advanced age, and greater comorbidity. The middle tertile (T2; mean UCR 19.05 ± 2.34) had the lowest observed mortality, but the tertile-based design does not establish a universal optimal interval or treatment target. Accordingly, UCR should be interpreted together with renal function, fluid balance, nutritional and catabolic context, and overall illness severity rather than as a direct measure of hydration.

Our results are consistent with reports that UCR carries prognostic information across related critical-illness phenotypes. Higher UCR has been associated with mortality in septic shock, cardiogenic shock, and trauma-related ARDS (33–35), while UCR has also been linked to AKI risk (22). These studies support the interpretation of UCR as an integrated marker of perfusion, renal handling, and catabolic stress rather than a disease-specific causal factor. The present study extends this literature to VAP and identifies a non-linear pattern, but direct comparison is limited by differences in case mix, outcomes, and covariate adjustment. The persistence of the T3 association after KDIGO and non-renal SOFA adjustment suggests that the observed high-UCR association was not explained solely by measured AKI stage; however, the shared creatinine component prevents a causal or mechanistic interpretation. Moreover, UCR produced only a small, statistically uncertain AUC increase beyond SOFA and no AUC gain beyond SAPS II, so incremental clinical utility was not established.

This study used a large critical-care database and applied multivariable and non-linear analyses. Several limitations should be emphasized. First, the retrospective single-center design precludes causal inference. Second, although KDIGO stage was reconstructed from creatinine, urine output, and CRRT, urine-output documentation over a complete 24-h window was available for only 424 patients during the first ICU day, and the MIMIC implementation used CRRT rather than every renal-replacement modality; stage 3 may therefore be incompletely ascertained. Third, KDIGO and UCR share serum creatinine, SOFA contains renal criteria, and SAPS II contains serum urea. The KDIGO-conditioned Cox models are alternative estimands, and incremental score comparisons may reflect part-whole overlap. Fourth, the cross-validated AUC differences were not statistically significant and no external validation was performed. Fifth, first-day albumin was available for only 513 patients (25.2%); the complete-case result may be affected by selective testing and cannot resolve catabolic confounding. APACHE II, active bleeding, protein intake, nitrogen balance, prealbumin, and tubular injury markers remained unavailable. Finally, the observed low-risk middle tertile is cohort-specific and should not be interpreted as a universal optimal range.

## 5 Conclusion

In critically ill patients with VAP, the highest admission UCR tertile was associated with higher 30-day mortality, and the overall pattern was non-linear. The low-UCR tertile was not independently associated with mortality after full adjustment. The high-UCR association persisted in the KDIGO and non-renal SOFA sensitivity model, but UCR did not significantly improve cross-validated AUC beyond SOFA or SAPS II. UCR should therefore be interpreted as an associative risk marker rather than a causal treatment target or an established incremental prediction tool; external validation is required.

## Data Availability

Publicly available datasets were analyzed in this study. The data that support the findings of this study are available from MIMIC-IV. Access to the database can be obtained through PhysioNet at https://mimic.mit.edu/. Researchers wishing to access the MIMIC-IV database must complete the required training and agree to the data use agreement available on the PhysioNet website.
